# Rules for biocatalyst and reaction engineering to implement effective, NAD(P)H-dependent, whole cell bioreductions

**DOI:** 10.1016/j.biotechadv.2015.08.006

**Published:** 2015-09-03

**Authors:** Regina Kratzer, John M. Woodley, Bernd Nidetzky

**Affiliations:** a Institute of Biotechnology and Biochemical Engineering, Graz University of Technology, Petersgasse 12/I, 8010 Graz, Austria; b CAPEC-PROCESS Research Center, Department of Chemical and Biochemical Engineering, Technical University of Denmark, Søltofts Plads Building 229, 2800 Kgs. Lyngby, Denmark

**Keywords:** Chiral alcohol, Decision tree for bioreduction development, Design of *Escherichia coli* whole cell catalysts, Limitations of whole cell reductions, Cost analysis, Scale-up

## Abstract

Access to chiral alcohols of high optical purity is today frequently provided by the enzymatic reduction of precursor ketones. However, bioreductions are complicated by the need for reducing equivalents in the form of NAD(P)H. The high price and molecular weight of NAD(P)H necessitate *in situ* recycling of catalytic quantities, which is mostly accomplished by enzymatic oxidation of a cheap co-substrate. The coupled oxidoreduction can be either performed by free enzymes in solution or by whole cells. Reductase selection, the decision between cell-free and whole cell reduction system, coenzyme recycling mode and reaction conditions represent design options that strongly affect bioreduction efficiency. In this paper, each option was critically scrutinized and decision rules formulated based on well-described literature examples. The development chain was visualized as a decision-tree that can be used to identify the most promising route towards the production of a specific chiral alcohol. General methods, applications and bottlenecks in the set-up are presented and key experiments required to “test” for decision-making attributes are defined. The reduction of *o*-chloroacetophenone to (*S*)-1-(2-chlorophenyl)ethanol was used as one example to demonstrate all the development steps. Detailed analysis of reported large scale bioreductions identified product isolation as a major bottleneck in process design.

## 1. Introduction

**Fig. 1:**
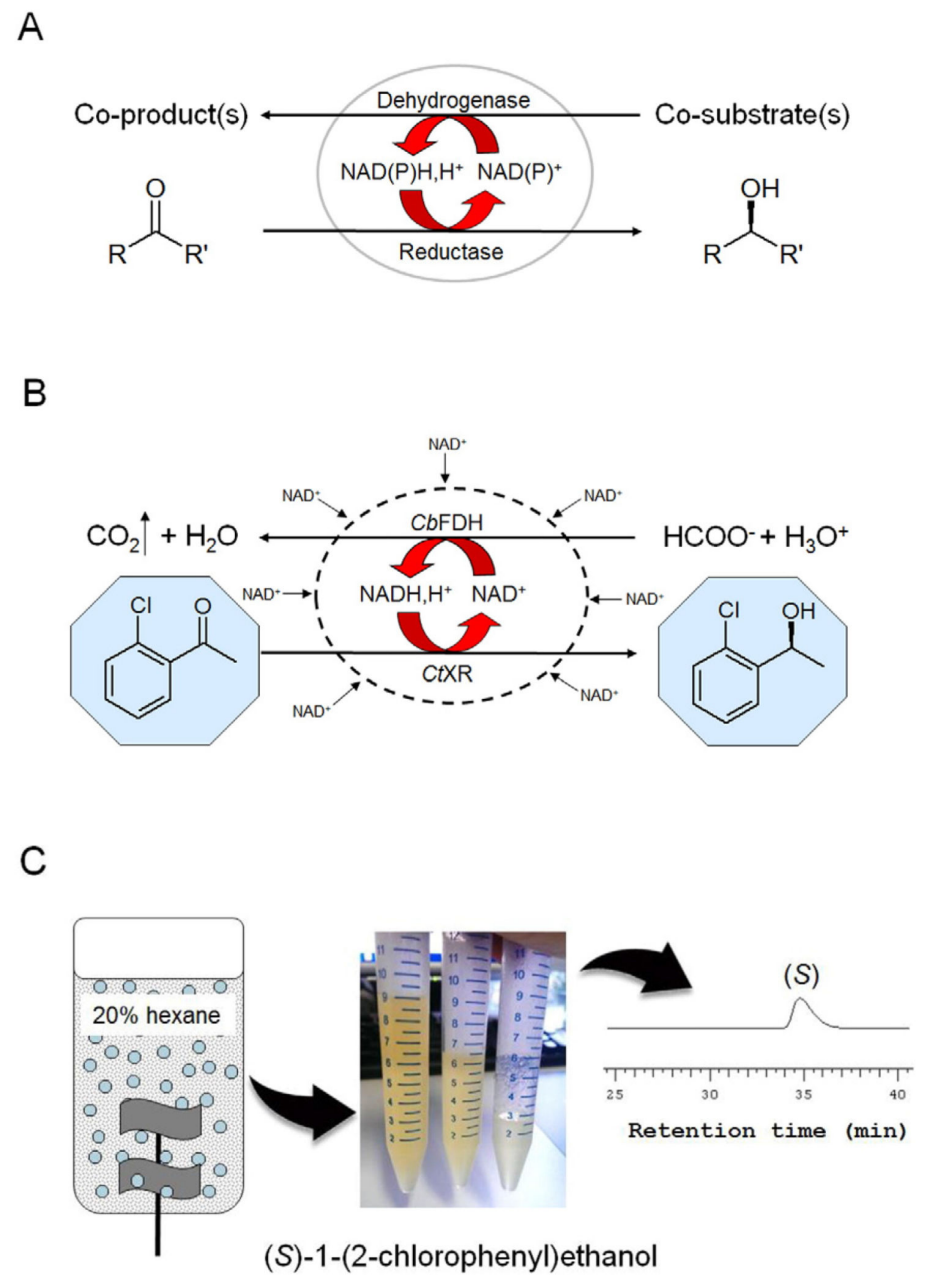
General scheme of bioreductions catalyzed by free enzymes or whole cells (gray oval indicates the cell envelope) (A). Whole cell reduction of *o*-chloroacetophenone catalyzed by recombinant *E. coli* based on *Ct*XR and *Cb*FDH (the dashed oval line depicts cell permeabilization, the blue hexagons illustrate *isss* and *is*pr by a water immiscible co-solvent). (B). Scheme of the multiphasic *o*-chloroacetophenone bioreduction at 0.5-L scale. The reaction was performed in a stirred tank reactor with pH and temperature control (gray points depict the biomass, blue drops show the hexane phase extracting *o*-chloroacetophenone and (*S*)-1-(2-chlorophenyl)ethanol). The three tubes show the extracted (*S*)-1-(2-chlorophenyl)ethanol that was obtained per batch (20 g) and that was further analyzed by chiral HPLC (C).

Numbers one, two, three and nine out of the 10 top-selling drugs in history are non-peptidic, enantiopure molecules (data from October 2013; Nixon, 2013) and chiral compounds will, as reckoned by analysts, still have a prominent position on blockbuster drug lists by 2020 (Brown, 2014). Single-enantiomer pharmaceuticals are typically administered in optical purities of 98% e.e. and above (Pollard and Woodley, 2007). Such enantiomeric purities are best obtained from enzyme-catalyzed reactions. Hence, there is a strong drive to implement biocatalytic steps into synthetic routes towards many pharmaceutical products (Wohlgemuth, 2007). Enantiopurity is generally obtained either by synthesizing specifically one enantiomer or resolving a racemic mixture. The quest for synthetic efficiency naturally favors asymmetric synthesis of one enantiomer from an achiral precursor over chiral resolution of a racemic mixture (Federsel, 2006; Straathof et al., 2002; Trost, 1991). The most often exploited enantioselective biotransformation in industry is the reduction of a ketone precursor into an optically pure alcohol (Pollard and Woodley, 2007). Enzymes catalyzing carbonyl reduction are mostly dependent on the reduced form of the coenzyme NAD(P). However, stoichiometric addition of NAD(P)H(>700 g/mol) is neither technically nor economically feasible, thus requiring the *in situ* recycling of catalytic quantities. Coenzyme regeneration is generally accomplished by the enzymatic oxidation of a cheap co-substrate. The coupled oxidoreduction is either performed by cell-free oxidoreductases or by whole cell systems (Fig. 1A). A pre-requisite for both systems is the selection of an enantioselective reductase. Oxidoreductase properties, the decision between cell-free and whole cell reduction system and reaction design complexity affect the bioreduction efficiency. A key task therefore in implementing a new bioreduction is the selection of the most efficient strategy among a multitude of possibilities.

### 1.1. Scope of the review

**Fig. 2:**
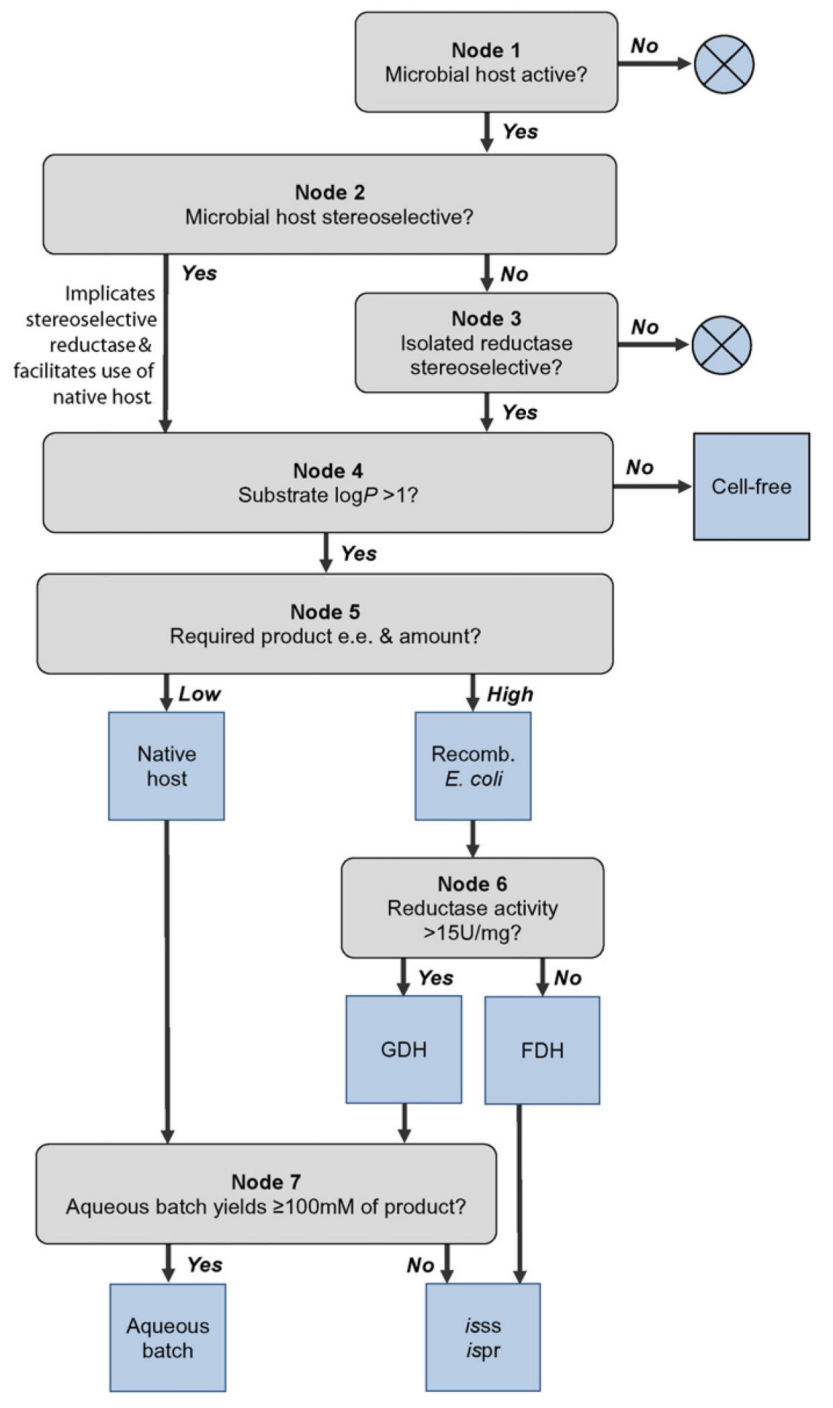
Decision tree for the set-up of bioreductions. Rounded rectangles represent decision rules, blue squares show process solutions, inefficient branches are terminated by blue circles. (Process options for cell-free bioreduction systems are not further developed.)

It is the aim of the present review to outline the design of whole cell bioreductions and critically scrutinize decisions along the development chain. We thereby introduce a graphical tool that helps to identify a strategy for the most effective production of a specific alcohol (Fig. 2). The depicted decision tree consists of seven nodes covering screening for active microbes, reductase selection, the decision between a cell-free and whole cell reduction system, coenzyme recycling alternatives and process options. Decisions are guided by the properties of the substrate and the requirement for optical purity and amount of product (Fig. 2). Decision rules are based on a small number of biocatalytic reactions that are thoroughly described in the scientific literature. Bottlenecks along the development cycle of bioprocesses are presented in detail. Finally scale-up aspects that are specific for whole cell bioreductions are discussed. Clearly, coverage of all scientific literature in the respective area is beyond the scope of this manuscript.

## 2. Development chain of bioreductions

### 2.1. Enzyme sources

The decision to use biocatalysis in synthetic chemistry is mainly justified by the superior enantioselectivity enzymes provide in comparison to chemo-catalysts. Enantioselectivity is hence the key criterion for enzyme selection in the production of chiral synthons (Kita et al., 1999; Pollard and Woodley, 2007). The wide abundance of reductases in nature renders screening of strain libraries or literature searching for the best fitting candidate oftentimes more cost and time effective than enzyme engineering.

#### 2.1.1. Screening of microbial hosts

The criterion for strain selection is primarily the whole cell activity towards the target substrate (Fig. 2, node 1). An enantiopure product proves the existence of one or more enantioselective reductases. In this case, the native strain might be directly applied in the production of a limited amount of product (Fig. 2) in particular in the early stages of process development (Pollard and Woodley, 2007). An active strain that shows low or no enantioselectivity harbors either enzymes that are unselective, enzymes with opposite enantioselectivities or both. The so-called “reductase background” of native hosts is strongly strain and substrate dependent. *o*-Chloroacetophenone, as an example, is barely utilized as a substrate by abundant reductases and is hence converted into the enantiopure alcohol by reductase-rich microorganisms like *Pichia stipitis*, *Candida tenuis* or *Candida pseudotropicalis* (Gruber et al., 2013; Xie et al., 2006). The ability to reduce, for instance, α-keto esters is common among reductases. Bioreductions of an ethyl benzoylformate by *C. tenuis* and *P. stipitis* cells resulted in low ee values of 65 and 80%, respectively (Gruber et al., 2013). Previous efforts to overcome contrasting reductase activities included adjusting the substrate concentration, use of additives to selectively inhibit one or more competing enzymes and genetic knockout approaches (Kaluzna et al., 2004). In practice, these strategies are often only partially successful and the more straightforward strategy in these cases is to investigate the enzyme(s) in its isolated form. Stereoselectivities of whole cells or isolated enzymes are analyzed by chiral chromatography of corresponding reduction products (Fig. 2, node 2, node 3).

### 2.2. Catalyst level

Stereoselective enzymes can generally be used in cell-free form or as whole cell catalysts. The cell envelope shields enzymes from the reaction medium and thereby increases enzyme stability but may also decrease reaction rate. The utilization of free reductases, on the contrary, minimizes mass transfer limitations but exposes the enzyme directly to adverse compounds of the reaction mixture. Hence, whole cells and free enzymes show opposite characteristics with respect to catalyst activity and lifetime. The way in which the total turnover of the catalyst (i.e. the product of catalyst activity and lifetime) is affected when whole cells are used instead of free enzymes, is case specific.

The cell envelope constitutes a barrier to the surrounding medium and therefore, transfer of hydrophilic compounds in and out of the cell requires specific transporter systems (Daugelavičius et al., 2000; Kell et al., 2015). Consequently, bioreductions of hydrophilic substrates that are harmless to free enzymes might yield higher product concentrations when oxidoreductases are directly applied as free enzymes. Mädje et al. (2012) have previously reported on a two-fold higher xylitol concentration when free enzymes were used instead of recombinant *Escherichia coli* in the bioreduction of xylose. Expression of a transporter protein is a further option. The co-expression of a transporter protein facilitated glucose uptake by an *E. coli* whole cell catalyst and increased mannitol production >10-fold (Kaup et al., 2004). Hydrophobic molecules enter the cell by slow diffusion through the lipopolysaccharide cell wall and subsequent partition into the cell membrane (Daugelavičius et al., 2000). Whole cells turned out as the catalyst of choice in cases of fast enzyme deactivation by hydrophobic substrates and products (Schmölzer et al., 2012). The cell envelope provides on the one hand protection of intracellular enzymes while on the other hand it is permeable enough for hydrophobic substrates to keep the reaction going. The bioreduction of ethyl 4-chlorobutanoate to ethyl (*S*)-4-chloro-3-hydroxybutyrate is one route towards the chiral side chain of the blood cholesterol lowering Atorvastatin, the top selling drug of all time (Nixon, 2013; Thayer, 2006). Ethyl (*S*)-4-chloro-3-hydroxybutyrate synthesis has been thoroughly optimized and product concentrations of 0.8 and 2.6 M were reported for reductions using free enzymes and whole cells, respectively (Kizaki et al., 2001; Shimizu et al., 1990). The low hydrophobicity of the substrate (log*P* 0.23; CAS database https://scifinder.cas.org) permits economic production using either free enzymes or whole cells (note the additional complication of substrate hydrolysis). Likewise, efficient production of L-*tert*-leucine by transaminases has been reported using free enzymes and whole cells (Tufvesson et al., 2011). These examples indicate that the hydrophobicity of a substrate can be used to choose the preferable form of the biocatalyst, i.e. free enzyme or whole cells. These examples suggest that hydrophilic substrates (log*P* ^<^ 0) are best converted by free enzymes and hydrophobic substrates (log*P* > 1) by whole cells. Additionally, substrates that show log*P* values between approximately 0 and 1 are efficiently converted by both whole cells and free enzymes. We used substrate log*P* ^<^ 1 as criteria for a cell-free bioreduction system (Fig. 2, node 4; the possibility to use whole cells or free enzymes for substrates with log*P* values between 0 and 1 is not indicated).

#### 2.2.1. Native hosts

Both recombinant and native microorganisms have been previously employed as bioreduction systems. The application of native strains shortens the process development time by avoiding multiple steps of strain construction (Pollard and Woodley, 2007). However, products obtained from bioreductions using native strains most often show lower enantiopurity and yields, compared to reductions catalyzed by recombinant strains of *E. coli* (Gruber et al., 2013; Yasohara et al., 1999; Kizaki et al., 2001). Usability of the native strain, either employed as cell-free or whole cell systems, depends therefore on the actual requirement for product purity and amount (Fig. 2, node 5).

#### 2.2.2. Recombinant E. coli

*E. coli* is easy to manipulate and has been shown to accumulate recombinant protein to a level of up to 55% of the soluble cell protein (Tishkov and Popov, 2006) or, as inclusion bodies, >50% of the total cellular protein (Baneyx and Mujacic, 2004). Recombinant *E. coli* is hence most often used as a “designer-bug” in whole cell bioreductions (Goldberg et al., 2007; Gröger et al., 2006; Kataoka et al., 2003). NAD(P)H-dependent carbonyl reductases transfer a hydride from the coenzyme to the carbonyl and protonate the nascent alcoholate. The reaction is hence consuming a hydride from NAD(P)H and a proton from the aqueous phase. A highly expressed reductase is, however, not optimally supplied with reduced coenzyme by the *E. coli* inherent metabolism (Kataoka et al., 1997). The expression of a second oxidoreductase to recycle NAD(P)H (Fig. 1A) is especially important under harsh reaction conditions that lead to elevated maintenance metabolism (Blank et al., 2008). Maximum use of the cell capacity is made when intracellular reductase and dehydrogenase activities are balanced against each other (Mädje et al., 2012). The most often exploited dehydrogenases in order to recycle NAD(P)H are formate and glucose dehydrogenases (FDHs, GDHs). Most FDHs are NAD^+^-specific; NADPH recycling by formate oxidation requires the application of a mutant FDH (Tishkov and Popov, 2006). In contrast, glucose dehydrogenases display dual specificities for NAD^+^ and NADP^+^. Both enzymes catalyze an irreversible reaction and thereby drive coupled reactions to completion. FDHs oxidize formate to carbon dioxide in a pH-neutral reaction. The overall oxidoreduction is consuming a proton as shown in Fig. 1B (Neuhauser et al., 1998). GDHs abstract a hydride and a proton from glucose to form NAD(P)H, H^+^ and glucono-δ-lactone. The overall reaction, when coupled to a carbonyl reductase, is pH neutral. The co-product glucono-δ-lactone is spontaneously hydrolysed leading to gluconate (Tishkov and Popov, 2006). FDHs display specific activities of 4 to 10 U/mg (EC 1.2.1.2; www.brenda-enzymes.org) while glucose dehydrogenases are among the most active dehydrogenases with activities of approximately 300 to 500 U/mg (EC 1.1.1.47; www.brenda-enzymes.org). So why not use GDH in all cases? NAD(P)H recycling by FDH, despite the enzyme’s low activity, displays three advantages. First, the molecular weight of formate (HCOONa) is 2.6-fold lower than that of glucose. Use of formate is hence more atom and cost economic. Secondly, the product of the reaction, carbon dioxide, is innocuous and evaporates from the reaction. Third, the required pH-titration by addition of formic acid provides an elegant way of pH-titration and co-substrate supply in a single step (Neuhauser et al., 1998). The reaction mixture is hence devoid of large amounts of co-substrate or co-product throughout reaction and product isolation when FDH is used for coenzyme recycling. Therefore, reductases with relatively low specific activities are generally coupled to FDHs in order to benefit from the process advantages that FDHs provide (Mädje et al., 2012). Indeed, the high specific activities of GDHs are hardly exploited when coupled to reductases with low specific activities and are hence co-expressed with highly active reductases to enable a fast intracellular oxidoreduction (Gröger et al., 2006; Kizaki et al., 2001). The decision between FDH and GDH as coenzyme recycling enzyme is therefore best based decided on the specific reductase activity. Reductases with specific activities of less than approximately 15 U/mg are best co-expressed with FDHs and reductases with higher specific activities coupled to GDHs (Fig. 2, node 6).

#### 2.2.3. Limitations of whole cell catalysts

##### 2.2.3.1. Mass transfer over cell wall and membrane

Whole cells usually show substantial activity losses when compared to their corresponding cell-free extracts (Alphand et al., 2003; Gruber et al., 2013; Rundbäck et al., 2012). Gruber et al. reported maximally 7% of the cell-free activity when recombinant *E. coli*, *Saccharomyces cerevisiae* and native *C. tenuis* cells were used for the reduction of *o*-chloroacetophenone (Fig. 1B). The loss of more than 90% activity is mainly ascribed to mass transfer limitations over the cell wall leading to a low intracellular concentration of (co)substrate. Effective substrate and co-substrate supply in bioreductions, as indicated in Fig. 1B, therefore require cell permeability to hydrophobic substrates and products, as well as hydrophilic co-substrates and co-products. Lipid bilayers are permeable to hydrophobic molecules although diffusion from the media into the cytoplasm is reduced by hydrophilic compounds in the cell wall. Charged or hydrophilic molecules, on the contrary, can only cross using porins, which are narrow channels for ions and small hydrophilic nutrients (Daugelavičius et al., 2000). Formate and glucose, the two most often used co-substrates in bioreductions based on *E. coli*, are taken up by native transporter systems of the host (Sawers, 2005). Glucose uptake in *E. coli* involves coupled transport and phosphorylation. The resulting glucose 6-phosphate is, however, not a substrate of GDH. An efficient glucose supply for GDH-catalyzed oxidation in *E. coli* therefore requires either engineering of the glucose uptake system or cell membrane permeabilization. Co-expression of the facilitator-type transporter GLF (glucose facilitator) from *Zymomonas mobilis* has been previously shown to increase the glucose import rate and decouple phosphorylation (Schroer et al., 2007).*E. coli* permeabilization using the peptide antibiotic polymyxin B sulfate (PMB) led to an improvement of up to 2-fold bioreduction as reported by Schmölzer et al. (2012). PMB shows a mainly locally disruptive effect on cell wall integrity in contrast to cell permeabilization by solvents, detergents or salts (reviewed by Chen, 2007).

The interaction of PMB with bacterial membranes starts with binding to the lipopolysaccharide (LPS) outer layer, thereby disturbing the LPS packing and promoting its own uptake (“self-promoted” uptake). In a second step, PMB induces the fusion of outer as well as cellular membranes to form envelope-crossing, ion permeable pores (Daugelavičius et al., 2000). The disordered LPS layer facilitates faster cell permeation by hydrophobic molecules while the formed pores connect the cytosol directly to the cell exterior and enable uptake of charged or hydrophilic compounds. Cell wall porosity leads also to diffusional loss of intracellular coenzymes and hence requires addition of NAD(P)(H) to reaction mixtures (Chen, 2007).

##### 2.2.3.2. Catalyst stability

Hydrophobic compounds generally show a toxic effect towards microbial cells by partition into cellular membranes. Accumulation of hydrophobic molecules results in swelling, permeabilization and finally loss of membrane integrity. Cell breakdown is estimated at membrane concentrations of around 300 to 400 mM of accumulated molecules. Partition of a hydrophobic compound into membranes is proportional to (1) its concentration in the aqueous phase and (2) its membrane-aqueous phase partition coefficient (De Bont, 1998). A relationship between the partitioning of a compound between the membrane and water (log*P*_M/W_) and its log*P* value has been previously reported by Sikkema et al. (1994) (Eq. (1)):

(1) $$\log P_{M∕W}=0.97\cdot \log P-0.64.$$

Eq. (1) allows predictions to be made about the toxicity of hydrophobic compounds based on their log*P* values. Highly hydrophobic molecules with log*P* values >4 accumulate in membranes but will not reach a high membrane concentration due to their low water solubility. In contrast, molecules with log*P* values between 1 and 4 are more water soluble and still partition into membranes, thus the achieved membrane concentration will be relatively high (De Bont, 1998). The question arises whether cells disintegrate prior to enzyme deactivation or enzymes get deactivated in the cell due to increasing concentrations of toxic molecules? The results obtained by Schroer et al. (2007) strongly suggest a deactivation mechanism with enzyme inactivation prior to cell disintegration. Two *E. coli* catalysts based on the same reductase but differing in the mode of coenzyme recycling were compared in terms of process stability. The alcohol dehydrogenase from *Lactobacillus brevis* *(Lb*ADH) was co-expressed with FDH or GDH in *E. coli* BL21 (DE3). Coenzyme recycling was either accomplished by formate oxidation with FDH or by glucose oxidation with GDH. The results indicated a strong dependence of catalyst stability on the expressed enzymes. The whole cell catalyst based on GDH showed better performance in the reduction of acetoacetate. The obtained catalyst half-life was at least 4-fold higher as compared to whole cell catalysts co-expressing FDH. GDHs are clearly preferable in continuous bioreduction processes with recombinant *E. coli* due to higher process stabilities of GDHs compared to FDHs (Schroer et al., 2007).

### 2.3. Reaction level

Eixelsberger et al. (2013) have previously interpreted the efficiency of a bioreduction to mean the final product concentration in the reaction mixture. Indeed, as a rule of thumb, product concentrations below ~100 mM generally render bioreduction processes uneconomic. Especially for whole cell bioreductions that exploit FDH for coenzyme recycling the use of *in situ* extraction of substrate and product into a water-immiscible, second phase is required to reach product concentrations of ≥100 mM (Schmölzer et al., 2012). On the contrary, product concentrations of 1 to 1.8 M have been reported for aqueous batch reductions with *E. coli* strains co-expressing GDH and a catalytic reductase (Ema et al., 2008; Gröger et al., 2006; Kataoka et al., 2003). The requirement for a second phase in bioreductions that are based on GDHs depends on the process stability of the carbonyl reductase. Therefore, aqueous batch reductions that yield product concentrations below 100 mM call for the application of a second phase in order to achieve economic product concentrations (Fig. 2, node 7).

#### 2.3.1.1. In situ *substrate supply (isss) and product removal* (is*pr)*

The addition of a water-immiscible phase to a stirred tank reactor containing the reaction mixture does not seem to be a complex task. The choice and amount of added solvent is, however, highly empirical and guidelines for the optimization of biphasic bioreductions are missing so far. The most important properties of suitable second phases are their extracting ability for substrate and product and their low solubility in the aqueous phase itself (log*P* > 4). The ideal second phase should provide an optimal substrate concentration in the aqueous phase and full extraction of the product. Preferred second phases show moderate to low boiling points (≤100 °C), are nontoxic and are readily available. The following literature examples for whole cell reactions might give a starting point for *in situ* substrate supply and product removal (*isss* and *is*pr) strategies of substrates and products with similar structures and log*P* values. Schmölzer et al. (2012) have reported on a ≥ 10-fold improved yield for an *E. coli* whole cell reduction of *o*-chloroacetophenone by the addition of 20% v/v *n*-hexane (Fig. 1B, C). The product concentration of an *E. coli* whole cell Baeyer–Villiger oxidation (bicyclo[3.2.0]hept-2-en-6-one to (−)-(1*S*,5*R*)-2-oxabicyclo[3.3.0]oct-6-en-3-one and (−)-(1*R*,5*S*)-3-oxabicyclo[3.3.0]oct-6-en-2-one) was 20-fold increased by the addition of 100 g/L hydrophobic resin (Optipore L-493; Alphand et al., 2003). Addition of 50% v/v *n*-butyl acetate to the *E. coli* whole cell reduction of 4-chloro-3-oxobutanoate to the corresponding *S*-alcohol led to a high product concentration of 3 M by prevention of substrate hydrolysis and biocatalyst protection. These examples indicate that efficient *isss* and *is*pr strategies are highly case-specific with respect to dissolution properties of the substrate (and the corresponding product), enzyme stabilities and enzyme kinetics.

### 2.4. Diagnostic parameters

Enantiomeric/regiomeric purity, product concentrations and total turnover numbers (expressed as gram product obtained per gram biocatalyst) are generally used to assess bioreactions (Pollard and Woodley, 2007; Straathof et al., 2002). However, the complexity of whole cell reductions requires yet further criteria to identify possible limitations. Table 1 lists seven additional parameters that describe whole cell reductions, but also provide scope for engineering (Gruber et al., 2013). The intracellular enzyme activity, whole cell activity, catalyst lifetime and extracellular substrate and product concentrations are directly measurable (for methods see Section 3). The comparison of intracellular and whole cell activity illustrates activity loss due to mass transfer limitations over the cell envelope and indicates low intracellular substrate concentrations. Cell permeability and intracellular substrate concentration are not directly measurable. Nevertheless, the effect of externally added NAD(P)(H) on reaction rates (or yields) gives qualitative information on cell wall integrity. Cell permeabilization is characterized by diffusional loss of NAD(P)(H), i.e. externally added NAD(P)(H) leads to higher reduction rates and yields. Intact cell membranes are impermeable for NAD(P)H and no effect is seen upon addition of coenzyme (Rundbäck et al., 2012). Low conversions that do not increase with added coenzyme indicate low intracellular (co)substrate concentrations. The combination of cell permeabilization and externally added coenzyme leads, in these cases, to higher reaction rates and yields (Table 1).

### 2.5. Practical use of the decision tree

Fig. 2 pictures the development chain of a bioreduction as a straight forward process from screening for stereoselective enzymes to reaction engineering. However, it is frequently more realistic to see the development process as a circle that might be progressed through a combination of both forward and backward steps. The development of a bioreduction is summarized in Table 2 using the example of *o*-chloroacetophenone reduction by yeast xylose reductase. Note that also in the present example the chronical sequence did not follow the development chain from top to bottom as depicted in Fig. 2. The starting point was the enzyme *Ct*XR already cloned into a standard pET vector (pET11a; Häcker et al., 1999) and not the native *C. tenuis* strain. The isolated enzyme showed high Prelog-type stereoselectivity in the reduction of aromatic ketones (Kratzer and Nidetzky, 2007; Kratzer et al., 2011) (node 3 in Fig. 2 and Table 2). Especially the reduction of *o*-chloroacetophenone provided a potentially interesting route towards (*S*)-1-(2-chlorophenyl)ethanol, a chiral key intermediate in the synthesis of a new class of chemotherapeutic drugs (Sato et al., 2009). The low stability of the free enzyme in the presence of *o*-chloroacetophenone and (*S*)-1-(2-chlorophenyl)ethanol indicated the application of a whole cell catalyst (Kratzer et al., 2011) (node 4, log*P*_*o*-chloroacetophenone_ 2.2, https://scifinder.cas.org). The native host was not a practical option for the required full conversion of 23 g starting material (Eixelsberger et al., 2013) (node 5, see also node 2) (Fig. 1C). The low specific *o*-chloroacetophenone reductase activity of 4.4 U/mg_protein_ suggested the co-expression of *Ct*XR with FDH in an *E. coli* host (Kratzer et al., 2011; Mädje et al., 2012) (node 6). The resulting recombinant whole cell catalyst was used in aqueous batch reductions of *o*-chloroacetophenone. Product concentrations of at most 98 mM indicated the application of *isss* and *is*pr to accomplish higher product concentrations (Kratzer et al., 2011; Schmölzer et al., 2012) (node 7).

## 3. Methods and applications

### 3.1. Screening of microbial strains

Screening for NAD(P)H-dependent reductase-activities in microbial strain libraries has been reported for a wide variety of ketone targets (e.g. Botes et al., 2002; Homann et al., 2004; Lavandera et al., 2008; Stampfer et al., 2004; Yasohara et al., 1999). The process of microbe selection for a specific reductase-activity is highly empirical, although a critical evaluation of relevant literature has very often been used to narrow down the choice of candidate strains (Botes et al., 2002; Homann et al., 2004; Yasohara et al., 1999). For instance, finding solvent-tolerant oxidoreductases was accomplished by screening microorganisms that are employed as bioremediators and can grow under extreme conditions (Lavandera et al., 2008). The general screening approach includes growing of strains according to established protocols and using them, with or without prior cell concentration, for conversions. Biomass concentrations expressed as gram cell dry weight per liter (g_CDW_/L) vary therefore from single digit to low three-digit values. The upper biomass concentrations used in biotransformations are generally limited by the viscosity of reaction mixtures to maximally ~200 g_CDW_/L (Shiloach and Fass, 2005). Addition of easily metabolizable compounds to the reaction mixture enables the cell to recycle the required NAD(P)H (Fig. 1). Glucose (1% w/v in Hall et al., 2006 and Homann et al., 2004; 4% in Yasohara et al., 1999), 2-propanol (10% v/v in Lavandera et al., 2008 and Stampfer et al., 2004) and glycerol (10% in Botes et al., 2002; 20% Homann et al., 2004) have been previously reported as co-substrates in screening protocols. The reaction mixtures are usually buffered to pH-values of 7.0 ± 0.5. The use of lyophilized cells allows, besides long-term storage of biocatalysts, improved substrate and product transfer through partially permeabilized membranes (Hall et al., 2006; Lavandera et al., 2008; Stampfer et al., 2004). Culture screens have been accomplished in micro-centrifuge tubes (Botes et al., 2002; Stampfer et al., 2004), 24 well plates (Homann et al., 2004; Stampfer et al., 2004) or shaken flasks (Homann et al., 2004). General reaction conditions are 30 °C and shaking for 8 to 48 h prior to analysis.

### 3.2. Enzymes from databases

The high abundance of NAD(P)(H)-dependent reductases in nature (Kaluzna et al., 2004) and the need of industry for chiral alcohols (Pollard and Woodley, 2007) led to the gathering of reductases and exploration of their product patterns, enantioselectivities, biochemical and kinetic data (reviewed in Matsuda et al., 2009). The most straightforward strategy to find a suitable reductase might therefore start from a search for the required alcohol on SciFinder (database for references, substances and reactions; http://www.cas.org/products/scifinder). Amino acids sequence and biochemical characteristics of enzyme matches can be further found in the BRENDA database (http://www.brenda-enzymes.org/), gene sequences in PubMed (http://www.ncbi.nlm.nih.gov/pubmed). Enzymes with similar protein or DNA sequence can be identified by an online BLAST search using the originally match as query (http://blast.ncbi.nlm.nih.gov/Blast.cgi). In the absence of an enzyme match the search for an alternative enzyme that produces a structurally closely related alcohol is suggested. Possible hits might catalyze the required reaction as an additional activity; for instance enzymes from the superfamily of aldo-keto reductases (https://www.med.upenn.edu/akr/) are well known for their ability to convert a broad spectrum of ketones (Ni et al., 2011). Engineering of the enzyme by directed evolution provides a method to engineer oxidoreductases without the knowledge of structure-function relationships. Requirement is a suitable screening method to select large numbers of mutant enzymes. High throughput screening methods to engineer oxidoreductases with regard to stability, catalytic activity and substrate specificity have been previously reviewed (Johannes et al., 2006). The availability of a structure facilitated engineering of the cofactor preference (Petschacher et al., 2005; Tishkov and Popov, 2006). Furthermore, fine-tuning of enantioselectivity by enzyme engineering based on enzyme-substrate docking has been reported previously (Musa et al., 2009; Zhang et al., 2009). In the rare case that no reductase is found that catalyzes the target reaction; the search for a ‘catalophore’ (three-dimensional constellation of functional groups in the active site) in structural databases might be used for the identification of novel enzymes as recently published by Steinkellner et al. (2014).

### 3.3. Analytics

Chiral GC with standard flame ionization detectors (FID) provide the required resolution and sensitivity and have been previously used for sufficiently volatile compounds, i.e. up to an analyte boiling point that is slightly beneath the temperature limit of the chiral column, typically 220-230 °C (Botes et al., 2002; Lavandera et al., 2008; Stampfer et al., 2004). Chiral HPLC has been used either coupled with refractive index (RI) detectors for molecules that are UV transparent or with UV detectors that measure UV absorption of the analytes (Homann et al., 2004; Schmölzer et al., 2012; Yasohara et al., 1999). Minimal analyte concentrations that are detectable in GC/FID and HPLC coupled to UV detectors are in the micromolar range. Detection limits are approximately one to two orders of magnitude higher when RI detectors are used. If standard material of alcohols is not available, neither as enantiomer nor as racemate, it is oftentimes relatively easy to obtain the racemate of the target alcohols by chemical reduction of the ketone substrate using versatile reducing agents such as sodium borohydride (March, 1992). A comparison of optical rotation to reported chiroptical data allows determination of the absolute configuration in cases where standards of alcohol antipodes are missing. The synthesis of diasteriomeric *R*- and *S*-MTPA (α-methoxy-α-(trifluoromethyl)-phenylacetyl) esters is used to determine the absolute configuration of the product alcohol in rare cases where neither antipode standards nor chiroptical data are available. The relative proximity of the phenyl group in the R- and S-MTPA esters to side chains of the alcohol is seen as chemical shifts of the alcohol side chains in ^1^H NMR (Dale and Mosher, 1973).

Strains harboring the desired activity do not necessarily reflect the stereoselectivity of a single enzyme. Reductase-rich organisms express enzymes with overlapping substrate specificities and differing stereoselectivities. A characterization of the enzyme with respect to specific activity and stereoselectivity therefore requires isolation of the protein.

### 3.4. Reductase activities

Most reductases used in biocatalysis catalyze a hydride transfer from NAD(P)H to the carbonyl C-atom and a proton transfer to the arising alcoholate O-atom. Specific activities are determined by the spectrophotometrically measured NAD(P)H consumption in buffered, aqueous media. Reaction conditions should guarantee substrate concentrations below inhibitory levels and enzyme stabilities of at least 2 min. The substrate solubility in aqueous media already dictates the useful concentration range for initial rate measurements. Concentrations above the substrate solubility lead to dispersions and destabilize enzymes at the boundary surface. Furthermore, substrate availability depends on the amount of dissolved substrate because there is no direct substrate transfer from a second phase to the biocatalyst (De Bont, 1998). The enzyme activity measured at the highest dissolvable substrate concentration equals the maximally obtainable specific activity. Determination of initial rates at different substrate concentrations provides information on substrate inhibition and gives a first estimation of enzyme stability. The amount of purified protein needed is determined by the enzyme activity. As a rule of thumb, one unit of enzyme activity measured at a substrate concentration that allows the exact determination of the ee-value after reaction is sufficient. Generally, reductases that show specific activities below 1 U/mg are less suitable in bioreductions. The enzyme stability in the presence of substrates and products in millimolar concentrations might be very low but can always be increased by the use of whole cells and addition of a co-solvent from the range of a few minutes to several hours (Schmölzer et al., 2012). Determination of the Michaelis-Menten constant (*K*_m_), turnover number (*k*_cat_) and substrate and product inhibition constants are of immediate importance when the free enzyme in solution is used as a catalyst. The effective substrate and product concentrations (i.e. concentrations at the enzyme) in whole cell reductions differ substantially from bulk concentrations and are not easily measurable (Gruber et al., 2013). In these cases, the catalytic activity at very low substrate concentrations becomes more important.

### 3.5. Design of whole cell catalysts based on E. coli

Aside for mass transfer issues, the specific activity of an *E. coli* whole cell catalyst depends on the specific activities of the reductase, the dehydrogenase and the respective expression levels. Finely tuned co-expression of oxidoreductases is required to optimally exploit the used enzymes. Recombinant production of the two enzymes in single-expression experiments might give a first clue of how to design the co-expression strain. The amount of accumulated recombinant protein is easily estimated from polyacrylamide gel electrophoresis (PAGE) (Laemmli, 1970). Functional expression is best checked in initial rate measurements in the cell-free extract. Additionally, specific activities for reductase and dehydrogenase measured in their respective single-expression strains indicate already the need for up- or down-regulation in the design of co-expression strains. However, reductase and dehydrogenase activity ratios obtained from single-expression strains do not necessarily predict relative expression levels in co-expression strains. Expression-levels depend on the properties of the protein itself, but it is not unusual to observe a decline in expression from single- to co-expression. In principle, co-expression is either achieved from a duet vector carrying two expression units or from two different plasmids maintained in one strain.

#### 3.5.1. Duet vectors

Duet vectors provide equal gene dosage of reductase and dehydrogenase in the producer cell. Novagen (part of Merck4Biosciences) markets 5 different types of duet vectors. These vectors provide T7 promoters for high-level expression by IPTG induction and require an *E. coli* host containing a chromosomal copy of the T7 RNA polymerase gene (*E. coli* (DE3)). Duet vectors from Novagen differ in antibiotic resistance and replicon/copy number i.e. pETDuet-1 vector with ampicillin resistance and ColE1 replicon/copy number 40; pACYCDuet-1 with chloramphenicol resistance and P15A replicon/copy number 10–12; pCDFDuet-1 with streptomycin resistance and CloDF13 replicon/copy number 20–40; pRSFDuet-1 with kanamycin resistance and RSF1030 replicon/copy number > 100; pCOLADuet-1 with kanamycin resistance and COLA replicon/copy number 20–40) (Held et al., 2004). These vectors contain two multiple cloning sites (MCS1, MCS2) with almost identical upstream transcription and translation signals, i.e. promoter-, operator-sequences and ribosome binding sites (Held et al., 2003, 2004; Novy et al., 2002). However, duet vectors from Novagen are lacking a terminator after the MCS1 leading to transcriptional read through that destabilizes the corresponding mRNA (Sørensen and Mortensen, 2005). Hence, the gene inserted into the MCS2 is generally expressed more strongly than the gene cloned into the MCS1. The use of duet plasmids provides expression of two proteins with only one antibiotic without plasmid compatibility issues.

#### 3.5.2. Two plasmids

Maintenance of two different plasmids in a single *E. coli* cell requires compatible vectors. It is not possible to maintain two plasmids that use the same mechanism for replication in a single cell. The replication of one plasmid will be inhibited as a result of an RNA antisense mechanism and one plasmid will prevail while the other will disappear. Furthermore, plasmid segregation occurs when both plasmids carry the same antibiotic resistance gene, since antibiotic resistance needs only one plasmid for maintenance. Therefore, plasmids sharing origins of replication from the same incompatibility group or common antibiotic resistance genes should be avoided for co-expression (Tolia and Joshua-Tor, 2006). The major advantage of the two-plasmid strategy is a higher flexibility in the combination of reductases and dehydrogenases. For example, if a reductase is cloned in a pET-vector with GDH and FDH in pRSF-vectors, enzyme combinations can be easily changed by re-transformation, thus avoiding tedious re-cloning.

Generally, co-expression of two proteins is also dependent on factors such as the differences in the relative rate of transcription and translation, stabilities of mRNA and protein, codon usage bias and repeat structures. Selection of the most suitable expression host is the easiest way to overcome (some of) these problems. The protease deficient strain *E. coli* BL21 is the most widely used host for protein expression. BL21 (DE3) contains an IPTG inducible T7 RNA polymerase and is designed for the expression of target proteins under T7 promoter control. BL21 star (DE3) is the RNaseE deficient variant that promotes higher mRNA stability and protein yield. Rosetta and Rosetta 2 are BL21 (DE3) strains that supply tRNAs for rare codons in *E. coli*. Rosetta strains are also obtainable as derivatives that provide enhanced disulfide bond formation (Rosetta-gami) (Held et al., 2003). The simplest optimization strategy is the transformation of plasmids into several BL21 (DE3) derivatives and protein expression at varying induction temperatures.

Co-expression of *Ct*XR and *Candida boidinii* FDH (*Cb*FDH) has been previously optimized. These enzymes show specific activities of ~4 U/mg under reaction conditions, i.e. 10 mM *o*-chloroacetophenone (solubility limit) and 150 to 350 mM sodium formate. Single-expression of *Ct*XR and *Cb*FDH in *E. coli* BL21 (DE3) led to 730 U/g_CDW_ and 400 U/g_CDW_, respectively. Co-expression performed under equal induction conditions resulted in full expression of *Ct*XR but decreased *Cb*FDH expression to 85 U/g_CDW_ (Kratzer et al., 2011; Mädje et al., 2012). *Cb*FDH co-expression was investigated with regard to gene copy number, expression strain and induction temperature. An experimental set-up following a 2^3^ factorial design was used to evaluate the most relevant variables and possible interactions. All three main factors significantly influenced *Cb*FDH activity; the effect of interactions were of minor importance. The *Cb*FDH activity increased 3-fold to a level of 250 U/g_CDW_ when a high copy number plasmid, an expression strain with optimized codon usage and low induction temperature was used. The *Ct*XR activity was reduced by just 19% under these conditions. The combined expression of *Cb*FDH and *Ct*XR in Rosetta 2 (DE3) adds up to 31% of soluble protein in *E. coli* (20% *Ct*XR, 11% *Cb*FDH). The obtained percentage is relatively high considering the codon usage bias of *E. coli* and yeast. Especially Arg codons used in *Cb*FDH and *Ct*XR count among the specifically rare codons found in *E. coli*. The Arg codons AGA and AGG are ~10-times more frequently used in *Cb*FDH and *Ct*XR as compared to its natural occurrence in *E. coli*. Furthermore, *Cb*FDH carries a tandem rare Arg codon double repeat (Arg210, Arg211) (Rare Codon Calculator; http://nihserver.mbi.ucla.edu/RACC/). Ribosome stalling at the tandem rare codon double repeat might account for low *Cb*FDH expression at concurrent high *Ct*XR production. Throttling of *Ct*XR expression and hence Arg codon consumption led to an increase in *Cb*FDH co-expression (Mädje et al., 2012).

There is usually no requirement for optimized expression of GDHs from *Bacillus* sp. as in most cases the enzyme is coupled to a reductase with much lower specific activity. We have previously cloned a *Bacillus megaterium* glucose dehydrogenase (*Bm*GDH) in a standard pRSF-1 plasmid (T7/*lac* operon, kanamycin resistance, RSF origin) and co-transformed BL21 (DE3) with the pET11a (ampicillin, pBR322 origin of replication) carrying the *Ct*XR gene. The *Bm*GDH activity was 2700 U/g_CDW_, i.e. 10-fold higher as compared to the *Cb*FDH expression following optimization as previously described. The obtained amount of functional enzyme corresponded to ~2% of the soluble protein in *E. coli* under co-expression conditions (Kratzer et al., unpublished). Hence, activities of *Bm*GDH and *Ct*XR were balanced in the respective strain without prior expression optimization.

#### 3.5.3. Scale-up of biomass production from shaken flask to bioreactor

Replacement of the shaken flasks by a bioreactor was used to intensify biomass production. However, this is frequently accompanied by reduced expression of recombinant protein in *E. coli* (Choi et al., 2006). Therefore, the main objective should be to increase the cell concentration while maintaining high activities of both enzymes in the co-expression strain. The bioreactor production of the co-expression strain was optimized in a stepwise manner. First, the bioreactor cultivation in batch mode was optimized. The complex media in the shaken flask was replaced by a mineral media that was optimized with respect to its C/N ratio. The optimal C/N ratio for co-expression was determined as 4.8 g/g and was used in all further bioreactor cultivations. Ampicillin employed in shaken flask cultivations was replaced by its more stable analogue carbenicillin (Lapidus et al., 1976). Fed-batch cultivations were started as optimized batches with 40 g/L glucose. After glucose was nearly depleted, the temperature was reduced to 18 °C, 1 mM of IPTG was added and continuous feeding started (12 h, OD_600_ 25). The nutrient feed was a 15-fold concentrated solution of the batch media and was fed exponentially. After 41 h an optical density of 37 and enzyme activities of 650 and 124 U/g_CDW_ were obtained for *Ct*XR and *Cb*FDH, respectively. Activity per volume was 6-fold higher for FDH and more than 12-fold higher for *Ct*XR. In this way, biomass production cost was reduced by 90% compared to previous shaken flask cultivations (Eixelsberger et al., 2013).

### 3.6. Reaction

We have previously analyzed bioreductions of *o*-chloroacetophenone in terms of gram product obtained per gram cell dry weight. Productivities (sometimes referred to as biocatalyst yield) specified as g_product_/g_catalyst_ reflect the product of catalyst activity (g_product_/(g_catalyst_ · h)) and effective catalyst lifetime (h). For example, the half-life of «3 min for the isolated *Ct*XR in the presence of 100 mM *o*-chloroacetophenone is far too low for substantial product formation (Kratzer et al., 2011). In order to harness the enzyme’s high stereoselectivity, native and recombinant whole cell catalysts based on *Ct*XR were used in bioreductions of *o*-chloroacetophenone (Gruber et al., 2013). The comparison is briefly summarized in Table 3.

#### 3.6.1. Comparison of strains

Diagnostic parameters introduced in Table 1 are used to illustrate differences of native and recombinant whole cell catalysts in the reduction of *o*-chloroacetophenone (the comparison is briefly summarized in Table 3). The required biomass was cultivated under optimal induction and growth conditions. Recombinant *E. coli* was grown in shaken flasks as previously reported and reached OD_600_-values of 3 to 3.5 (Kratzer et al., 2011; Mädje et al., 2012). The native yeast *C. tenuis* (CBS 4435) was grown on xylose as the sole carbon source and harvested at an OD_600_ of ≤5. Older cultures with OD_600_-values of >10 or cultures that grew on glucose showed 90 to 95% lower xylose reductase activities (Gruber et al., 2013). Intracellular enzyme activities were determined in the cell-free extracts as described under 3.3. Reductase activities of ~ 370 U/g_CDW_ were obtained for *C. tenuis* cultures, ~50–90% of the corresponding values for recombinant *E. coli* strains (Table 3) (Kratzer et al., 2011; Mädje et al., 2012). *E. coli* biomass was re-suspended in potassium phosphate buffer (100 mM, pH 6.5), supplemented with 150 mM sodium formate (50 mM excess as compared to the ketone concentration) (Kratzer et al., 2011; Schmölzer et al., 2012). Yeast cells were likewise suspended in potassium phosphate buffer (50 mM, pH 5.5), supplemented with 200 mM glucose as co-substrate. Biomass concentrations were adjusted to OD_600_ of 103 and 115 for *E. coli* and native yeasts, respectively, equivalent to approximately 40 g_CDW_/L (Gruber et al., 2013). Fast deactivation of the biocatalyst by hydrophobic substrates required substrates to be added just before mixing was started (Schmölzer et al., 2012). Gentle mixing was accomplished on an end-over-end rotator (model SB3 from Stuart, VWR, Austria) at 30 rpm and 25 °C. Analysis of the reaction mixture after 24 h showed that (*S*)-1-(2-chlorophenyl)ethanol concentration was 15 mM with an optical purity of ≥99.9% e.e. when native *C. tenuis* was used as catalyst (Gruber et al., 2013). Final product concentrations of *E. coli* catalyzed whole cell reductions were between 16 and 65 mM (Table 3) (Kratzer et al., 2011; Schmölzer et al., 2012).

##### 3.6.1.1. Time course analysis

Conversion versus time plots give information on initial reduction rates (reductase activity of the whole cell) and allow estimations of catalyst lifetimes. The linear part of the time course curve, starting from time point zero, provides the initial rate. Most accurate and reproducible values are obtained when conversion between time points are low (10–20%) and at least two measuring points are in the linear range. Initial rates of 10 and ≤50 U/g_CDW_ were obtained for *C. tenuis* and recombinant *E. coli*, equal to 3 and 7% of *o*-chloroacetophenone reductase activities measured in respective cell-free extracts (Table 3).

The lifetime of the catalyst is the time from reaction start to the time point when product formation levels off (provided that the conversion is «100%). Lifetimes of four to five hours were estimated from time courses of 100 mM *o*-chloroacetophenone reduction for recombinant *E. coli* (Kratzer et al., 2011). Catalyst lifetimes, as obtained from a comparison of initial rates and product concentrations are ≥33 min for *E. coli* and ~38 min for *C. tenuis* (Table 3). The example of *o*-chloroacetophenone reduction by whole cells based on *Ct*XR therefore provides an extreme case of catalyst instability. The comparison of whole cell catalysts with respect to mass transfer limitations and catalyst lifetimes suggests higher activity and lower stability of the *E. coli* whole cell catalyst. Fast cell permeabilization might cause accelerated enzyme deactivation but also reduced mass transfer over the membrane leading to the observed low reproducibility of product concentrations and initial rates (Table 3).

##### 3.6.1.2. Catalyst stability

Incubation of biomass with substrate or product under conditions similar to batch reductions and determination of the remaining activities over time gives precise information on catalyst deactivation. We have previously incubated recombinant *E. coli* in the presence of 0–50 mM *o*-chloroacetophenone or 1-(2-chlorophenyl)ethanol (Schmölzer et al., 2012). 100 μL samples were taken over time and immediately diluted 20-fold with buffer prior to cell harvesting by centrifugation. Cells were lysed using B-Per (Bacterial Protein Extraction Reagent from Thermo scientific) and subsequently *Ct*XR and *Cb*FDH activities were assayed. Activities followed an exponential decay over time and half-lives were calculated. Reference values for *Ct*XR and *Cb*FDH incubated in buffer were determined to be 5 and 8 days, respectively. At substrate and product concentrations of 30 mM the half-lives of *Ct*XR and *Cb*FDH decreased to ≤1%, equal to 1 h. The presence of 50 mM 1-(2 chlorophenyl)ethanol had an especially negative impact on *Ct*XR and *Cb*FDH activities and decreased the whole cell catalyst half-life to 10 min (Schmölzer et al., 2012). A higher toxicity of the product, as seen in the bioreduction of *o*-chloroacetophenone, renders the application of substrate supply rather inefficient. In cases of higher substrate toxicity, feeding strategies have proved successful. Doig et al. (2002) have previously shown that the lactone substrate bicyclo[3.3.0]hept-2-en-6-one is inhibitory in whole cell Baeyer–Villiger oxidations at concentrations 10-fold lower than the products. The inhibitory effect of the lactone substrate was overcome by substrate feeding and *in situ* substrate supply.

#### 3.6.2. Reaction optimization

##### 3.6.2.1. pH

Enzymatic carbonyl reduction by NAD(P)H requires protonation of the nascent alcoholate by a proton donor i.e. an acidic amino acid in the active site. Plots of enzyme activity as a function of pH show that activities are constant beneath and decrease above a corresponding p*K*_a_ value. The obtained p*K*_a_ value most often reflects the p*K*_a_ value of the proton donor in the active site. Reductase p*K*_a_ values are in general between 7 and 8. Dehydrogenases catalyze the reverse reaction and show activity versus pH profiles that mirror the curves obtained for the reduction reactions. The corresponding p*K*_b_ values are again between 7 and 8 (Schlegel et al., 1998). Optimization of the reaction pH is an additional method to balance reductase and dehydrogenase activities in cases where the cell is suitably permeabilized. Schmölzer et al. (2012) have previously optimized the pH of the *o*-chloroacetophenone reduction using recombinant *E. coli* based on the oxidoreductase couple *Ct*XR and *Cb*FDH. They used 100 mM potassium phosphate buffer, which was adjusted to pH values of 6.2, 6.5, 7.0, and 7.5 in aqueous batch reductions of 100 mM *o*-chloroacetophenone. Yields in aqueous batch reductions of 100 mM *o*-chloroacetophenone increased 2-fold upon a pH shift from 7.5 to 6.2. The *o*-chloroacetophenone reductase activity measured in the cell-free extract doubled from 347 to 741 U/g_CDW_ in response to a pH shift from 7.5 to 6.2. The *Cb*FDH activity determined at pH 6.2 decreased by 25% compared to the value obtained at pH 7.5. The reflection of *Ct*XR-activity in reduction yields suggests equal extracellular and intracellular pH values and hence a high perforation level of the cell. The pH effect seen on yields of whole cell reductions might be not only due to an increased reductase activity but also to an increased natural formate uptake at acidic pH (Lü et al., 2011; Rossmann et al., 1991).Eixelsberger et al. (2013) reported a pH shift from 6.2 to 7.7 during the reduction of 300 mM *o*-chloroacetophenone in 100 mM potassium phosphate buffer. pH control by automated addition of 1 M H_3_PO_4_ increased the conversion from 92.6 to 97.0%.

##### 3.6.2.2. In situ substrate supply and product removal

There are essentially two strategies for testing different *isss* and *is*pr conditions. First, keeping a constant amount of catalyst per total reaction volume and secondly, keeping a constant amount of catalyst per aqueous phase volume. The first strategy facilitates comparison of various conditions based on total reaction volume whereas the second strategy might provide a more knowledge-based optimization procedure by keeping aqueous phase conditions constant. Both optimization strategies are simplified by preliminary assessment of substrate and product toxicity towards the whole cell catalyst (see section *Catalyst stability* under 3.6.1.2) separately from measurement of distribution coefficients in the absence of biomass.

Further optimization of the *o*-chloroacetophenone reduction by recombinant *E. coli* was started with the selection of suitable second phases. Kratzer et al. (2008, 2011) have previously shown that relatively polar organic solvents with log *P* < 4 are not well suited as second phases in *E. coli* based reductions. Therefore the common solvents hexane, heptane and dodecane covering a log *P* from 4.11 to 6.8 and the ionic liquid BMIMPF_6_ were selected. The substrate was pre-dissolved in the various solvents. Reactions were started by addition of *E. coli* cells suspended in buffered co-substrate solution. Overall concentrations of cells were 40 g_CDW_/L, substrate concentrations 100 or 200 mM and co-substrate concentrations exceeded substrate concentrations by 50 mM. Parallel experiments containing additionally either 0.5 mM NAD^+^, 36 μM polymyxin B sulfate or both were also made. The addition of NAD^+^ led to higher product yields when no co-solvent, hexane or heptane was used. No effect was seen when NAD^+^ was added to reaction mixtures containing dodecane or ionic liquid. Improved yields by the addition of NAD^+^ suggest either solvent or substrate permeabilization of the cell wall (Table 1). Controlled permeabilization with polymyxin B sulfate led to further yield improvements in reaction mixtures without co-solvent, with hexane or heptane. The absence of yield improvement by polymyxin B sulfate when dodecane or BMIMPF_6_ were used as second phases suggests that the substrate concentrations in the aqueous phase were too low (Table 1). The combination of externally added hexane, NAD^+^ and polymyxin B sulfate as well as a pH shift from 7.5 to 6.2 led to an almost 20-fold improved product concentration, compared to the standard, aqueous batch reduction. The conversion of 300 mM *o*-chloroacetophenone using 50 g_CDW_/L was scaled-up to 500 mL. Hexane (20% v/v) served as second phase during the reaction and was also used in subsequent product extraction. Reaction and product isolation were performed in a 1 L bioreactor using stirrer, pH and thermostatic control. The product (*S*)-1-(2-chlorophenyl)ethanol was isolated in a total yield of 86%(Fig. 1C) (Eixelsberger et al., 2013).

### 3.7. Scale-up of whole cell bioreductions

Each of the unit operations: cell production, bioreduction and product recovery constitutes a scale-up task with particular requirements. Cultivation of recombinant *E. coli* is generally benchmarked by the obtained recombinant protein per liter of growth medium (Schmid et al., 2001). High enzyme titers of up to 10 g/L were previously obtained from high cell density cultures (reviewed in Choi et al., 2006). One of the main problems in the scale-up of *E. coli* cultivation is acetate formation due to (locally) high glucose concentrations and oxygen transfer limitations. *E. coli* produces acetate as an extracellular co-product of the glucose overflow metabolism and, under anaerobic conditions, the mixed-acid fermentation. Acetate decelerates growth even at concentrations as low as 0.5 g/L, and it inhibits protein formation (Xu et al., 1999). Cell concentrations of up to 100 g_CDW_/L require hence bioreactors with high mixing efficiency, aeration with oxygen enriched air or pure oxygen, suitable nutrient feeding strategies and, furthermore, efficient cooling (Choi et al., 2006). The *k*_L_*a* value as a measure of gas–liquid distribution and mixing efficiency provides a useful scale-up criterion for *E. coli* cultivation. Optimized culture conditions obtained from microwell plates have previously shown to translate into 7.5 L and 75 L stirred tank reactors when *k*_L_*a* values were kept constant. Cell growth, protein expression and substrate consumption were accurately translated (Islam et al., 2008). Nutrient feeding with feedback control is widely employed for the high cell density cultivation of *E. coli*. pH value and oxygen concentration increase when the glucose becomes depleted. Controlling pH or dissolved oxygen and keeping it constant by feeding concentrated nutrient solution into the bioreactor provides a glucose concentration that is near zero. Overfeeding with glucose and concomitant acetic acid formation is thereby prevented. Process parameters like specific growth rates (pre- and post-induction), induction times and inducer concentrations individually affect expression of reductase and dehydrogenase in co-expression strains (Choi et al., 2006). The requirement for high and balanced expression of both activities represents an additional scale-up criterion in the biomass production (Mädje et al., 2012; Eixelsberger et al., 2013). Use of a high catalyst loading that is sufficient inexpensive (67 €/kg at 10 m^3^ cultivation volume, Tufvesson et al., 2011) might compensate for low cell activities but will hamper product isolation. A maximal catalyst loading of 50 g_CDW_/L was previously published for two liquid phase, whole cell bioreductions in order to limit product loss in downstream processing to ≤20% (Eixelsberger et al., 2013). The bioreduction was performed in a stirred tank reactor at 500 mL scale. Liquid–liquid dispersion was performed with a Rushton turbine at a stirrer speed of 500 rpm and a power input per unit volume of 1.19 W/dm^3^. The maintenance of constant interfacial area per unit volume was identified as key parameter for the scale-up of two liquid phase, whole cell biotransformations (3 to 75 L; Cull et al., 2002). Constant power input per unit volume was found to be the most suitable scale-up parameter. A minimal value of 0.38 W/dm^3^ was published for the scale-up of two liquid phase, whole cell biotransformations applicable to reactors with three-stage Rushton impeller system (Cull et al., 2002). However, a low catalyst concentration of ~3.5 g_CDW_/L, as used by the authors, might not be comparable to reaction mixtures with high catalyst loading. The formation of a (kinetically) stable emulsion when a catalyst concentration of 50 g_CDW_/L was used, as reported by Eixelsberger et al. (2013), might require further scale-up criteria. Though, the performance of a batch bioreduction in a standard bioreactor for the cultivation of bacteria or yeast will always be possible. The efficiency of product isolation in whole cell biocatalysis strongly relates to phase separation quality. Product separation from the biomass in large scale bioreduction/biooxidation mixtures include ispr onto beads (Vicenzi et al., 1997), liquid extraction of the product following the bioreduction (Baldwin et al., 2008), separation of the biomass from the product flow by a membrane and *in situ* product crystallization and product crystallization subsequent to the bioreduction (Buque-Taboada et al., 2006).

#### 3.7.1. Product isolation

Product extraction from reaction mixtures containing whole cells has been previously described as ‘downstream nightmares’ (Chen, 2007). Vicenzi et al. (1997) reported on a 300-L scale bioreduction of 3,4-methylene-dioxyphenyl acetone to the corresponding *S*-alcohol by *Zygosaccharomyces rouxii*. Substrate was *in situ* supplied on a hydrophobic resin with 500 μm particle size and the product likewise extracted from the aqueous phase onto the resin. A reactor (350 L) containing a filter on a shaft (33 rpm, pore size 100–200 μm) was used for the bioreduction and product isolation. Filter agitation provided a fluidized bed of resin and yeast cells with low-shear mixing during the reaction. Subsequent filtering retains beads on the screen and removes biomass; constant revolving of the screen impedes filter clogging by the biomass. The product was recovered in a yield of 85 to 90% by washing the resin with acetone. For a higher yield the diluted, aqueous phase containing the remaining product was passed through a bed of resin. Spontaneous phase separation of a Baeyer–Villiger oxidation at 200-L scale using a whole cell biocatalyst at an exceptionally low catalyst concentration of 1.5 g_CDW_/L is emphasized. The biotransformation was performed in aqueous medium with subsequent product extraction using 20% v/v ethylacetate. Spontaneous separation was accelerated by the addition of 15% w/v sodium chloride (Baldwin et al., 2008). Buque-Taboada et al. (2006) reviewed the removal of chiral alcohols from biotransformation by crystallization at industrial scale. The reduction of 4-oxoisophorone to levidione at 1.65 m^3^ was used as a base case by the authors. The reduction was performed in a stirred tank reactor coupled with an external crystallizer separated by an ultrafiltration membrane for cell retention. Crystallization was also used for the isolation of the product (*R*)-N-(2-hydroxy-2-pyridin-3-yl-ethyl)-2-(4-nitro-phenyl)-acetamide (100 g/L) from a large-scale whole cell bioreduction mixture. Downstream processing included dissolution of the precipitated alcohol by acidification, filtration of the catalyst *Candida sorbophila* and addition of base to crystallize the alcohol (Moore et al., 2007). Product isolation constituted a major cost factor in these examples and additional separation of unreacted starting material would further increase downstream processing cost. In particular at larger scale catalyst loading has to be optimized with respect to both conversion as well as product separation.

## Figures and Tables

**Table 1:** Diagnostic parameters of whole cell reductions.

Parameter	Assessed	Experiment
Stereoselectivity	Directly	Chiral HPLC, GC (see also Section 3.3)
Intracellular enzyme activity (activities)	Directly	Photometric measurement of NAD(P)(H) depletion (formation) in cell-free extracts (3.4)
Whole cell activity	Directly	Initial rates of whole cell reductions (3.6.1)
Activity loss (mass transfer limitation over cell wall)	Indirectly	Comparison of intracellular enzyme and whole cell activities (3.6.1)
Extracellular substrate, product concentrations	Directly	HPLC, GC (3.3)
Cell permeability	Indirectly	Effect of externally added NAD(P)(H) on reaction rates and yields (3.6.2)
Intracellular substrate, product concentrations	Indirectly	Effect of additional cell permeabilization on reaction rates and yields (3.6.1)
Whole cell catalyst lifetime under process conditions	Directly	Time course analysis of whole cell reductions (3.6.1)
	Indirectly	Comparison of whole cell activities and product concentrations (3.6.1)
Total turnover number	Indirectly	Gram product per gram catalyst (3.6)

**Table 2:** Development chain of *o*-chloroacetophenone reduction by *Ct*XR.

# of node/decision rule	Object to be examined	Answer	Decision
1 Is the microbial host active?	Native *C. tenuis*	Yes (370 U/g_CDW_) (Gruber et al., 2013)	Native host contains stereoselective reductase(*s*)
2 Is the microbial host stereoselective?	Native *C. tenuis*	Yes (>99.9% e.e.) (Gruber *et al.*, 2013)	Native host is suitable for product amounts of ~1–5 g
3 Is the enzyme stereoselective?	Isolated *Ct*XR	Yes (>99.9% e.e.) (Kratzer et al., 2011)	Keep enzyme
4 log*P*_substrate_ > 1?	*o*-Chloroacetophenone	Yes (log*P* = 2.2) (https://scifinder.cas.org)	Use whole cell reduction
5 Optical product purity & amount?	Product requirement	20 g (Eixelsberger et al., 2013)	Use recombinant *E. coli*
6 Reductase activity ≤ 15 U/mg?	*Ct*XR specific activity	Yes (4.4 U/mg) (Kratzer et al., 2011; Mädje et al., 2012)	Couple to FDH
7 Product concentration ≥ 100 mM?	Yields of aqueous batch reductions	No (16–98 mM) (Kratzer et al., 2011; Schmölzer et al., 2012)	Use 2^nd^ phase (*isss*, *is*pr)

**Table 3:** Systematic comparison of native and recombinant hosts based on *Ct*XR in whole cell reductions of *o*-chloroacetophenone.

Parameter	*C. tenuis* (Gruber et al., 2013)	*E. coli*
Stereoselectivity (ee %)	> 99.9 *S*	> 99.9 *S* (Kratzer et al., 2011)
Intracellular enzyme activity^a^ (U/g_CDW_)	370	420–730 (Kratzer et al., 2011; Mädje et al., 2012)
Whole cell activity^b^ (U/g_CDW_)	10	≤50 (Kratzer et al., 2011)
Activity loss of cell-free extract vs whole cell^c^ (%)	~97	~93
Product concentration^d^ (mM) Aqueous batch reduction	15	16–65^e^ (Kratzer et al., 2011; Schmölzer et al., 2012)
Whole cell catalyst lifetime^f^ (min)	~38	≥33 4–5 h^g^ (Kratzer et al., 2011)
Maximal total turnover number (g_product_/g_CDW_)	0.25	0.39

a Initial rates of 10 mM *o*-chloroacetophenone reduction in buffer.

b Initial rates of aqueous batch reductions with 100 mM *o*-chloroacetophenone.

c Comparison of initial rates is admissible despite differing substrate concentrations a 10 mM represents the solubility limit of *o*-chloroacetophenone.

d Substrate concentration was 100 mM *o*-chloroacetophenone.

e Results obtained without added NAD^+^ and without prior cell permeabilization.

f Estimated from whole cell activity and final product concentration.

g Taken from time course.
